# Self-perceptions of body weight status according to age-groups among Korean women: A nationwide population-based survey

**DOI:** 10.1371/journal.pone.0210486

**Published:** 2019-01-17

**Authors:** Boyoung Park, Ha Na Cho, Eunji Choi, Da Hea Seo, Sue Kim, Yeong-Ran Park, Kui Son Choi, Yumie Rhee

**Affiliations:** 1 Department of Medicine, Hanyang University College of Medicine, Seoul, Republic of Korea; 2 Department of Cancer Control and Population Health, Graduate School of Cancer Science and Policy, National Cancer Center, Goyang, Republic of Korea; 3 Department of Endocrinology and Metabolism, School of Medicine, Inha University, Incheon, Republic of Korea; 4 College of Nursing, Yonsei University, Seoul, Republic of Korea; 5 Department of Silver Industry, Kangnam University, Yongin, Republic of Korea; 6 Department of Internal Medicine, Endocrine Research Institute, Severance Hospital, Yonsei University College of Medicine, Seoul, Republic of Korea; Harvard T.H. Chan School of Public Health, UNITED STATES

## Abstract

While numerous studies have investigated body image, including body weight perception, most of which have focused on adolescents or young women, few studies have attempted to evaluate body weight perceptions in adult women according to age groups. This study was conducted to investigate the accuracy of self-perceived weight and actual body mass index (BMI) values among adult Korean women according to age. We used data from the 2016 Korean Study of Women’s Health Related Issues, a population-based, nationwide, cross-sectional survey. BMI was calculated from self-reported weight and height. Participants were asked to describe their body image by choosing one of the following descriptions: very underweight, underweight, about right, overweight, or obese. The proportions of women aged 20–29, 30–39, 40–49, 50–59, 60–69, and 70–79 years who underestimated their body weight relative to their actual BMI category were 12.6%, 15.1%, 22.2%, 34.0%, 45.6%, and 50.7%, respectively; those who overestimated their body weight comprised 18.7%, 17.8%, 14.3%, 10.8%, and 7.4%. In all BMI categories, the proportion of those who overestimated their weight status increased as age decreased, while those who underestimated their weight status increased as age increased. After adjusting for possible covariates, age was strongly associated with both underestimation and overestimation. The odds ratio for underestimating one’s weight status among women aged 70–79 yeas was 2.96 (95% CI: 2.10–4.18), and that for overestimation was 0.52 (95% CI: 0.35–0.79), compared to women aged 20–29 years. Age is the most important factor associated with weight perceptions among Korean women, affecting both underestimation and overestimation of weight status.

## Introduction

Obesity and overweight are emerging health issues worldwide, with global prevalences of 39% and 13%, respectively, in the year 2016 [1, 2]. Trends in the prevalence of obesity and overweight have been shown to differ between sexes, and gender disparities therein have been found to vary from region to region [3]. In general, the prevalence of obesity and overweight is higher in women than in men, especially in low-to-middle income countries [3, 4]. However, in developed countries, more men are overweight than women [3] and in developed Asian countries, the prevalence of obesity has substantially increased among men and decreased among women [5–7]. Social stigma against obesity and overweight may partly be responsible for this trend, which was found to be extremely high in developed countries [8] and in females residing in East Asian countries [9–11].

Obesity stigma appears to arise from a widespread belief that weight is entirely an individual’s responsibility and from a media preference for thinness, especially toward women [12, 13]. In Asian countries, preference for thinness, especially among women, has increased rapidly, more so than in Western countries [14]. According to age groups, prevalence of obesity tends to be low in young adult women, but high in older women [5, 7]. This trend might be influenced by different motivations toward weight control between age groups and more societal stigma toward obesity in younger women [15, 16]. Indeed, one study reported that older adults were strongly motivated by the health risks associated with excess weight when considering weight control, whereas younger adults were motivated by the desire to improve appearance, to participate in social activities, and to form relationships [17]. In addition, younger females appear to be more prominently influenced by an apparent emphasis on being thin in media [3, 18, 19], and being ultra-thin is often considered an ideal body image [20].

Body image has been found to greatly affect attitudes and behaviors toward weight control [21, 22]. Body image reflects an individual’s awareness of their body weight status (normal, underweight, or overweight) in relation to their actual body weight [23]. However, considering that obesity stigma, which appear to be more strongly felt by women, especially younger women [12, 13, 15, 16], and considering that the idealized image for young women is being thin [3, 18, 19], one could expect that the accuracy and direction of perceptions of body weight would differ according to gender and age groups. The proportion of women who perceive themselves as being overweight has been shown to be much higher than that for men, irrespective of their body mass index (BMI) status in all age groups [24]. In addition, perceptions of being overweight and weight control attempts were found to be most common among young women residing in Asian countries, compared to European, Mediterranean, or American countries [24]. Previous studies have indicated that the older people with overweight or obesity are more likely to underestimate their weight status than younger people [25–27]. Altogether, these findings could potentially explain the unique trend of decreasing obesity among women according to age in Asian countries [5–7].

While numerous studies have investigated body image as body weight perceptions in adolescents or young women [24, 28] and according to age groups [27, 29–31], few studies have attempted to evaluate body weight perceptions in regards to both underestimation and overestimation thereof. Accurate weight perception itself is important in terms of initialization of weight control, and inaccurate weight perception has been associated with unhealthy weight control in previous studies. In addition, misperceptions of weight can affect weight control behavior according to its direction: underestimation has been shown to be associated with healthy weight control behaviors, whereas overestimation has been found to be associated with more unhealthy weight control behaviors [27].

Thus, we deemed that perceptions of weight in relation to both under- and overestimation, as well as age, warrant study. Therefore, we aimed to examine self-perceptions of weight status among women according to age in a large, nationally representative sample in comparison to their actual BMI values, based on self-reported height and weight.

## Materials and methods

### Study participants

Data were collected from the 2016 Korean Study of Women’s Health Related Issues (K-Stori), a population-based, nationwide, cross-sectional survey undertaken to investigate health awareness and needs assessments according to stages in the life cycle of women (adolescence, 14–17 years; childbearing, 19–44 years; pregnancy and postpartum, 19–44 years; perimenopause, 45–64 years; and older adulthood, 65–79 years). Stratified multistage random sampling was applied to select the study participants in each life cycle. First, we stratified the sample according to 16 municipalities and age based on the 2010 Resident Registration Population data. The number of administrative districts was designated in proportion to their population size, and study areas were randomly selected. Then, five to eight households in each urban area and 10 to 12 households in each rural area were selected randomly. Trained interviewers then contacted individuals by going door-to-door and assessed their study eligibility. All eligible participants in the house were then surveyed using a standardized questionnaire through face-to-face interviews after obtaining informed consent from April 2016 to June 2016. For the adolescence period only, an online survey was conducted after obtaining informed consent from both the participant and their parents. The questionnaire surveyed self-reported health status, perceived risk of cancer, individual health behaviors, medical service use, health information, social support, violence, gender role, and socio-economic status. Of the 37,334 people who we attempted to contact for an interview, 15,084 completed the interview. The survey response rate was 40.4%. This study was approved by the institutional review board of the National Cancer Center, Korea (NCC2016-0062). Details on the K-Stori have been described elsewhere [32].

Of the 15,084 study participants, we excluded 6,178 women from the adolescent group (defined by the K-Stori as ages 14–17 years) and the pregnancy and postpartum periods, as well as those who were younger than 20 years of age in the childbearing period, from the analysis due to a different definition of obesity among women younger than 20 years [33] and the existence of significantly greater misperceptions of height and weight among pregnant women [34]. Finally, a total of 8,906 women who provided information on their height, weight, and weight perceptions were included in the analysis.

### Data collection

Anthropometric information was collected by self-reported height and weight data. BMI was calculated therefrom, and participants were categorized according to criteria for Asians [35] or generally applied definitions for the Korean population [5, 36, 37] using BMI cut offs of <18.5 kg/m^2^ (underweight), <23 kg/m^2^ (normal), ≥23 kg/m^2^ (overweight), and ≥25 kg/m^2^ (obese).

Perceived weight status was assessed by asking participants to describe their body image according to the following list: very underweight, underweight, about right, overweight, or obese. We categorized their responses into four categories: underweight (combining very underweight and underweight), normal, overweight, and obese. Then, we compared the participants’ perceived weight status with their actual BMI category, and divided participants into three groups accordingly: correct estimation (perceived weight status corresponded with their actual BMI category), underestimation (perceived weight status was below their actual BMI category), and overestimation (perceived weight status was above their actual BMI category). Women were categorized into 10-year interval, age groups: 20–29, 30–39, 40–49, 50–59, 60–69, or 70–79 years. Considered covariates included residential area (urban as *eup* and *myeon* or rural area as *dong*) according to administrative divisions of Korea, education level (≤elementary school, middle school, high school, or college or more), marital status (single, married, or divorced/widowed), household income (< $2000/month, $2000-3999/month, or ≥ $4,000/month), smoking and alcohol drinking status (never or ever), and perceived health status (healthy, normal, or unhealthy).

### Statistical analysis

Descriptive statistics of socio-demographic characteristics of the study participants according to age groups were compared using the chi-square test for contingency tables and one-way analysis of variance (ANOVA) for continuous variables. A weighted Kappa coefficient was applied to assess the accuracy of perceptions of body weight: agreement between perceived weight status and actual BMI category. For each actual BMI decile, proportions of perceived weight statuses were calculated according to the age groups. Additionally, the proportions of correct estimation, underestimation, and overestimation according to age groups were also presented for each actual BMI category (underweight, normal, overweight, and obese). Multinomial logistic regression analysis was conducted to investigate whether individual age groups were associated with underestimation or overestimation and to explore factors independently associated with misperception of weight status. All *P*-values < 0.05 were considered statistically significant. Analyses were performed using R software (version 3.2.2).

### Ethical consideration

The study was approved by the institutional review board of the National Cancer Center, Korea (NCC2016-0062). An approved study description was provided to all eligible participants. If the subjects agreed to participate in the study after reading the study description, participants were asked to provide written informed consent. For adolescents, because they were under 18 years of age, parental consent was obtained at the time of recruitment, or a parental consent form was sent to the adolescents’ homes and the survey was carried out after confirming consent. Women who were pregnant or who had recently given birth were allowed to consult with their spouse or partner and discuss their participation in the survey.

## Results

Table 1 shows the socio-demographic characteristics of the study participants by age groups. Except for residential area, the distributions of education levels, marital status, household income, smoking status, drinking status, and perceived health status were all significantly different between age groups (all *P* < 0.001).

**Table 1:** Socio-demographic characteristics of respondents according to age groups.

	Total	20–29 years	30–39 years	40–49 years	50–59 years	60–69 years	70–79 years	P-value*
	(N = 8,906)	(N = 1,211)	(N = 1,195)	(N = 1,242)	(N = 1,502)	(N = 1,756)	(N = 2,000)	
Residential area								
Urban	7077 (79.5%)	970 (80.1%)	954 (79.8%)	997 (80.3%)	1200 (79.9%)	1380 (78.6%)	1576 (78.8%)	0.786
Rural	1829 (20.5%)	241 (19.9%)	241 (20.2%)	245 (19.7%)	302 (20.1%)	376 (21.4%)	424 (21.2%)	
Education								
≤Elementary school	1923 (21.6%)	4 (0.3%)	3 (0.3%)	6 (0.5%)	65 (4.3%)	520 (29.6%)	1325 (66.2%)	<0.001
Middle school	1231 (13.8%)	5 (0.4%)	7 (0.6%)	24 (1.9%)	162 (10.8%)	565 (32.2%)	468 (23.4%)	
High school	2832 (31.8%)	210 (17.3%)	289 (24.2%)	613 (49.4%)	933 (62.1%)	601 (34.2%)	186 (9.3%)	
College or more	2920 (32.8%)	992 (81.9%)	896 (75.0%)	599 (48.2%)	342 (22.8%)	70 (4.0%)	21 (1.0%)	
Marital status								
Single	1447 (16.2%)	1070 (88.4%)	313 (26.2%)	27 (2.2%)	11 (0.7%)	13 (0.7%)	13 (0.6%)	<0.001
Married	6043 (67.9%)	130 (10.7%)	854 (71.5%)	1150 (92.6%)	1370 (91.2%)	1427 (81.3%)	1112 (55.6%)	
Divorced or widow	1416 (15.9%)	11 (0.9%)	28 (2.3%)	65 (5.2%)	121 (8.1%)	316 (18.0%)	875 (43.8%)	
Household income								
< $2,000/month	2331 (26.2%)	105 (8.7%)	57 (4.8%)	49 (3.9%)	119 (7.9%)	645 (36.7%)	1356 (67.8%)	<0.001
$2,000–3,999/month	3582 (40.2%)	422 (34.8%)	613 (51.3%)	556 (44.8%)	712 (47.4%)	795 (45.3%)	484 (24.2%)	
≥ $4,000/month	2993 (33.6%)	684 (56.5%)	525 (43.9%)	637 (51.3%)	671 (44.7%)	316 (18.0%)	160 (8.0%)	
Smoking								
Never	8444 (94.8%)	1098 (90.7%)	1121 (93.8%)	1182 (95.2%)	1449 (96.5%)	1686 (96.0%)	1908 (95.4%)	<0.001
Ever	462 (5.2%)	113 (9.3%)	74 (6.2%)	60 (4.8%)	53 (3.5%)	70 (4.0%)	92 (4.6%)	
Drinking								
Never	2059 (23.1%)	142 (11.7%)	185 (15.5%)	209 (16.8%)	285 (19.0%)	526 (30.0%)	712 (35.6%)	<0.001
Ever	6847 (76.9%)	1069 (88.3%)	1010 (84.5%)	1033 (83.2%)	1217 (81.0%)	1230 (70.0%)	1288 (64.4%)	
Perceived health status								
Healthy	4583 (51.5%)	989 (81.7%)	887 (74.2%)	780 (62.8%)	803 (53.5%)	696 (39.6%)	428 (21.4%)	<0.001
Normal	2929 (32.9%)	201 (16.6%)	271 (22.7%)	399 (32.1%)	539 (35.9%)	713 (40.6%)	806 (40.3%)	
Unhealthy	1394 (15.7%)	21 (1.7%)	37 (3.1%)	63 (5.1%)	160 (10.7%)	347 (19.8%)	766 (38.3%)	

* Chi-square P-value

As age increased, mean BMI significantly increased (*P* < 0.001, Table 2). The prevalence of both underweight and normal BMI categories decreased as age increased; meanwhile, those for the overweight and obese BMI categories increased from 10.9% in women aged 20–29 years to 60% in women aged 70–79 years. The proportion of women who perceived themselves as overweight or obese also increased as age increased, from 20.7% in women aged 20–29 years to 44.1% in women aged 70–79 years, indicating that older women, overall, tended to underestimate their body weight relative to their actual BMI category, especially those in the obese category. Additionally, as shown in Table 2, we found that 54.9% of women perceived their body weight status correctly, while 33.0% underestimated and 12.1% overestimated their body weight status. The proportions of women aged 20–29, 30–39, 40–49, 50–59, 60–69, and 70–79 years who underestimated their body weight relative to their actual BMI category were 12.6%, 15.1%, 22.2%, 34.0%, 45.6%, and 50.7%, respectively; those who overestimated their body weight comprised 18.7%, 17.8%, 14.3%, 10.8%, and 7.4%.

**Table 2:** Weight status based on self-reported height and weight, weight perceptions, and correctness of weight perceptions among respondents according to age groups.

	Total	20–29 years	30–39 years	40–49 years	50–59 years	60–69 years	70–79 years	P-value*
	(N = 8,906)	(N = 1,211)	(N = 1,195)	(N = 1,242)	(N = 1,502)	(N = 1,756)	(N = 2,000)	
Body Mass Index								
Mean (SD)	22.6 ± 2.7	20.5 ± 2.1	21.3 ± 2.3	22.1 ± 2.3	22.9 ± 2.3	23.6 ± 2.4	23.7 ± 2.7	<0.001
Underweight	400 (4.5%)	164 (13.5%)	91 (7.6%)	51 (4.1%)	29 (1.9%)	19 (1.1%)	46 (2.3%)	<0.001
Normal weight	4777 (53.6%)	915 (75.6%)	888 (74.3%)	790 (63.6%)	746 (49.7%)	685 (39.0%)	753 (37.6%)	
Overweight	2057 (23.1%)	86 (7.1%)	129 (10.8%)	260 (20.9%)	455 (30.3%)	564 (32.1%)	563 (28.1%)	
Obese	1672 (18.8%)	46 (3.8%)	87 (7.3%)	141 (11.4%)	272 (18.1%)	488 (27.8%)	638 (31.9%)	
Weight perception								
Underweight	879 (9.9%)	188 (15.5%)	141 (11.8%)	120 (9.7%)	879 (7.1%)	120 (6.8%)	204 (10.2%)	<0.001
Normal weight	4637 (52.1%)	772 (63.7%)	710 (59.4%)	655 (52.7%)	4637 (49.9%)	838 (47.7%)	913 (45.6%)	
Overweight	2942 (33.0%)	219 (18.1%)	296 (24.8%)	414 (33.3%)	2942 (37.9%)	682 (38.8%)	762 (38.1%)	
Obese	448 (5.0%)	32 (2.6%)	48 (4.0%)	53 (4.3%)	448 (5.2%)	116 (6.6%)	121 (6.0%)	
Correctness of weight perceptions								
Underestimation	2935 (33.0%)	153 (12.6%)	181 (15.1%)	276 (22.2%)	511 (34.0%)	800 (45.6%)	1014 (50.7%)	<0.001
Correct estimation	4893 (54.9%)	831 (68.6%)	801 (67.0%)	789 (63.5%)	829 (55.2%)	806 (45.9%)	837 (41.9%)	
Overestimation	1078 (12.1%)	227 (18.7%)	213 (17.8%)	177 (14.3%)	162 (10.8%)	150 (8.5%)	149 (7.4%)	

* Chi-square P-value

Table 3 lists the agreement between BMI classification based on self-reported height and weight and self-perceptions of body weight status. The weighted Kappa coefficients indicated moderate agreement between perceived weight status and BMI categories in women aged less than 60 years (over 0.5) and fair agreement in women aged 60 years or more (around 0.4).

**Table 3:** Agreement of body mass index classification based on self-reported height and weight with self-perceptions of weight.

Body mass index	Self-perception of weight (%)				
	Underweight	Normal weight	Overweight	Obese	Weighted Kappa
20–29 years					
Underweight	6.8	6.4.6	0.3	0.0	0.560
Normal weight	8.8	55.2	11.0	0.6	
Overweight	0.0	1.7	5.0	0.4	
Obese	0.0	0.3	1.8	1.7	
30–39 years					
Underweight	4.0	3.3	0.3	0.0	0.542
Normal weight	7.5	53.1	12.1	1.6	
Overweight	0.2	2.1	8.0	0.5	
Obese	0.1	0.9	4.4	1.9	
40–49 years					
Underweight	2.7	1.4	0.0	0.0	0.588
Normal weight	7.0	44.2	11.0	1.4	
Overweight	0.0	6.3	14.3	0.4	
Obese	0.0	0.8	8.1	2.4	
50–59 years					
Underweight	1.9	0.4	0.0	0.0	0.502
Normal weight	7.5	23.6	5.1	1.4	
Overweight	0.7	14.7	12.2	0.5	
Obese	0.1	6.9	20.8	4.1	
60–69 years					
Underweight	0.7	0.4	0.0	0.0	0.441
Normal weight	5.5	26.1	5.8	1.6	
Overweight	0.5	16.0	14.9	0.8	
Obese	0.2	5.2	18.2	4.2	
70–79 years					
Underweight	1.9	0.4	0.0	0.0	0.459
Normal weight	7.5	23.6	5.1	1.4	
Overweight	0.7	14.7	12.2	0.5	
Obese	0.1	6.9	20.8	4.1	

**Fig 1:**
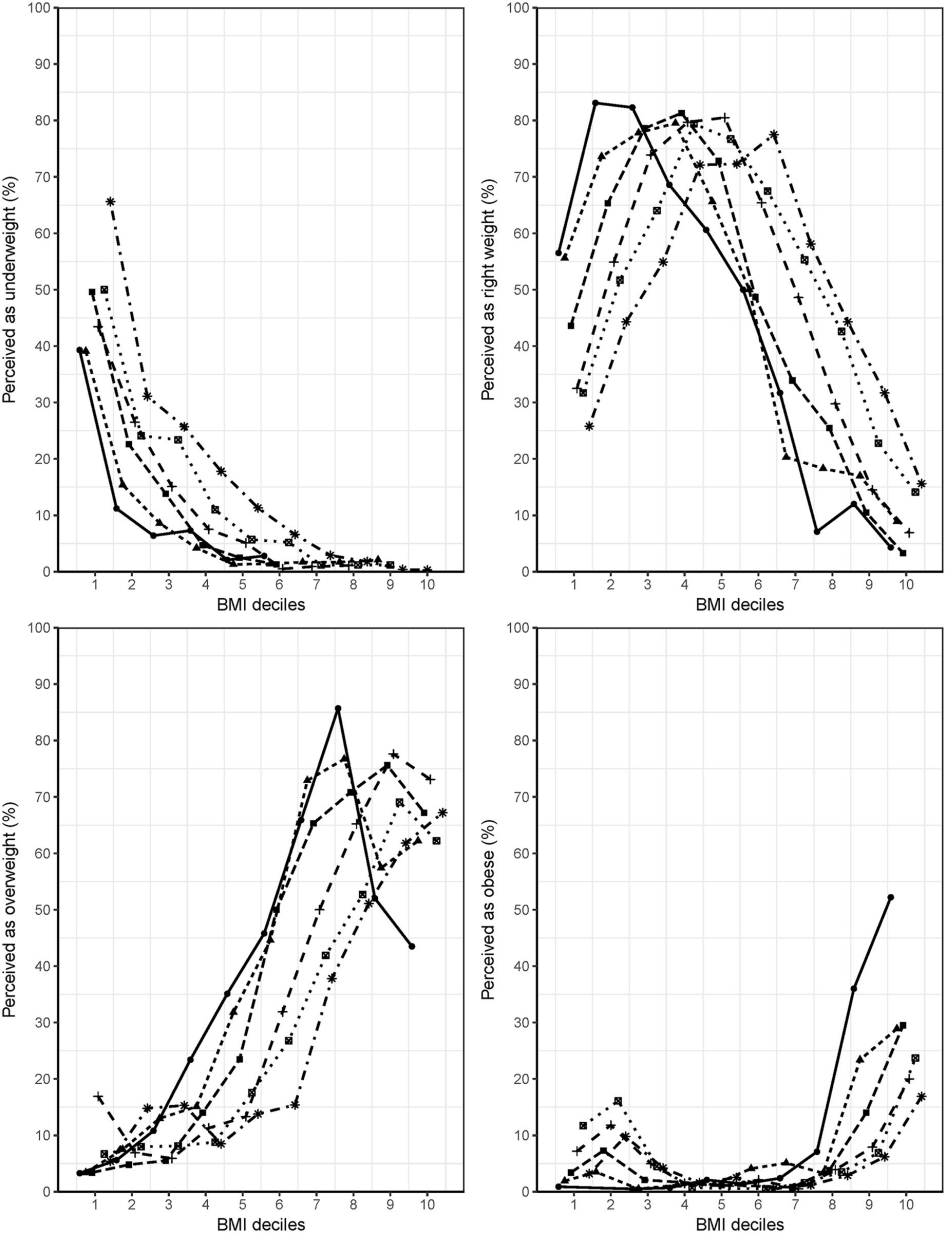
Prevalence of underweight, right weight, overweight, and obese perceptions according to body mass index deciles for age groups.

Fig 1 charts the prevalence of perceived weight status across BMI deciles for each age group. In all BMI deciles, the proportion of women who perceived themselves as being underweight was highest in the age group of 70–79 years, and the proportion decreased as age decreased. A reverse trend was observed for women who perceived themselves as being overweight or obese, showing the highest proportion in the age group of 20–29 years. In women who perceived themselves as normal or overweight, the distribution was right shifted as age increased, suggesting that general underestimation of body weight occurred in older age groups and overestimation in the younger age group.

**Fig 2:**
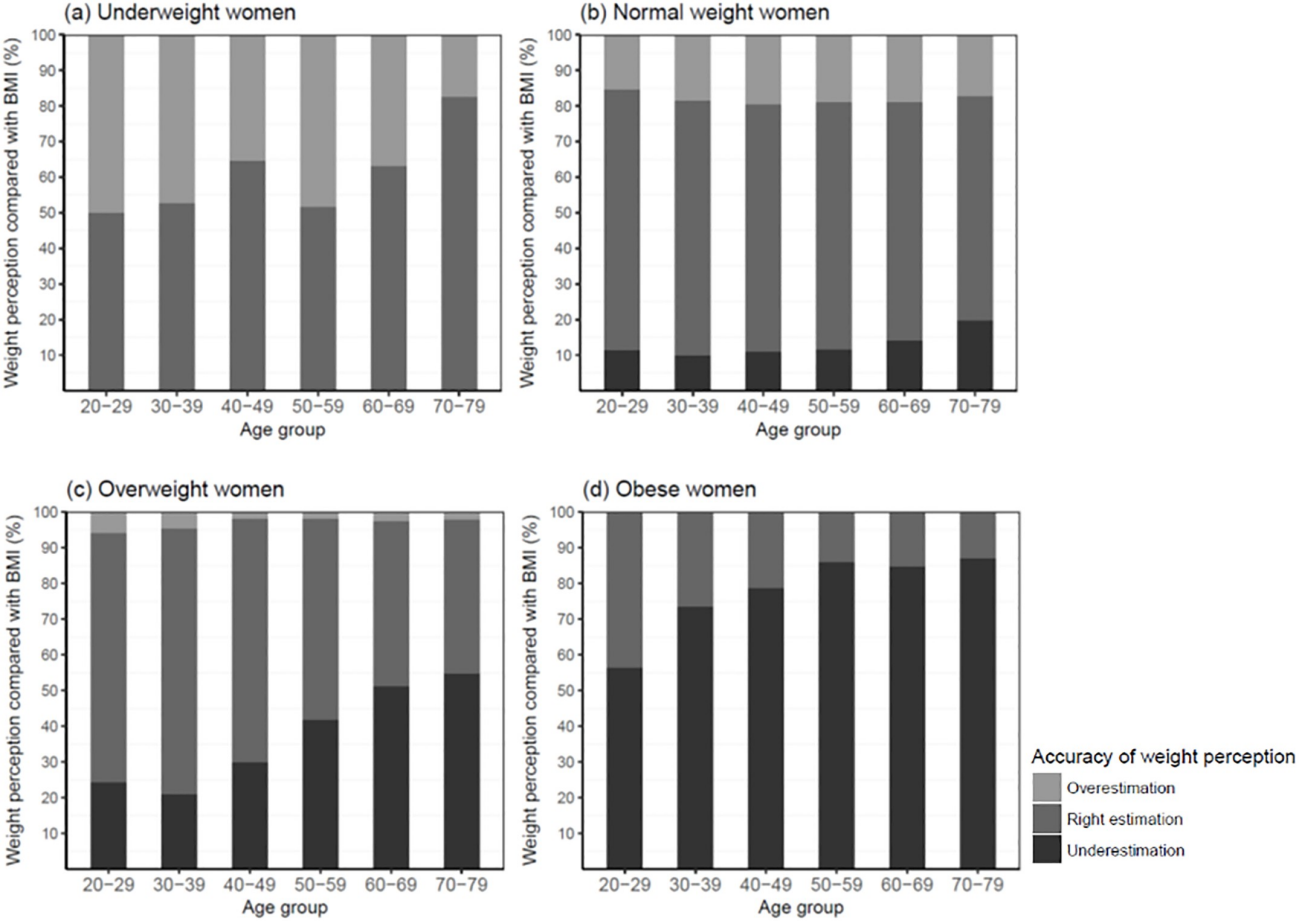
Accuracy of body weight perceptions according to age groups.

Fig 2 presents the accuracy of body weight perceptions according to BMI categories and age groups. In general, only 51.2% of underweight, 68.8% of normal, 54.0% of overweight, and 17.3% of obese women perceived their weight status correctly. In all BMI categories, the proportion of those who overestimated their weight status increased as age decreased, while those who underestimated their weight status increased as age increased. Even in the underweight category, 48.8% of women overestimated their weight status and it was decreased as age increased. Underestimation was higher among obese women as well, of which 82.7% underestimated their weight status. The overall proportion of women who underestimated their weight states increased as age increased with 87.1% in women aged 70–79 years.

Table 4 outlines the results of our multinomial logistic regression analysis of factors associated with the accuracy of women’s perceptions of their actual body weight. After controlling for other covariates, age was strongly associated with both underestimation and overestimation. Compared to women aged 20–29 years, older women were significantly more likely to underestimate their body weight status, but less likely to overestimate it. The odds ratios for underestimation in women aged 40–49, 50–59, 60–69, and 70–79 years were 1.53, 2.32, 2.91, and 2.96 (95% CI: 1.11–2.10, 1,69–3.19, 2.10–4.04, and 2.10–4.18) and those for overestimation were 0.81, 0.66, 0.58, and 0.52 (95% CI: 0.59–1.11, 0.47–0.92, 0.40–0.84, and 0.35–0.79), compared to women aged 20–29 years. Other potentially associated factors were education and household income for underestimation: women with higher levels of education or with a household income level of $2,000–3,999/month were less likely to underestimate their weight status. Women whose perceived health status was normal or unhealthy were more likely to overestimate their weight status, compared to healthy women.

**Table 4:** Multinomial logistic regression results for factors associated with misperception of weight among females.

	Underestimation		Overestimation	
	OR (95% CI)	P-value*	OR (95% CI)	P-value*
Residential area				
Urban	1		1	
Rural	0.97 (0.86–1.10)	0.637	0.99 (0.84–1.17)	0.928
Age group (years)				
20–29	1		1	
30–39	1.14 (0.85–1.52)	0.373	1.02 (0.78–1.33)	0.893
40–49	1.53 (1.11–2.10)	0.009	0.81 (0.59–1.11)	0.195
50–59	2.32 (1.69–3.19)	<0.001	0.66 (0.47–0.92)	0.014
60–69	2.91 (2.10–4.04)	<0.001	0.58 (0.40–0.84)	0.004
70–79	2.96 (2.10–4.18)	<0.001	0.52 (0.35–0.79)	0.002
Education				
≤Elementary school	1		1	
Middle school	0.86 (0.74–1.01)	0.074	1.18 (0.88–1.57)	0.273
High school	0.71 (0.59–0.85)	<0.001	1.05 (0.76–1.43)	0.778
College or more	0.46 (0.37–0.58)	<0.001	0.89 (0.62–1.26)	0.508
Marital status				
Single	1		1	
Married	1.08 (0.83–1.41)	0.575	0.92 (0.71–1.20)	0.527
Divorced or widow	1.07 (0.80–1.44)	0.649	0.97 (0.69–1.37)	0.885
Household income				
< $2,000/month	1		1	
$2,000–3,999/month	0.86 (0.75–0.99)	0.039	0.99 (0.79–1.24)	0.941
$4,000/month	0.88 (0.75–1.04)	0.143	1.09 (0.85–1.39)	0.504
Smoking				
Never	1		1	
Ever	0.99 (0.78–1.24)	0.898	1.30 (1.00–1.70)	0.052
Drinking				
Never	1		1	
Ever	1.04 (0.93–1.16)	0.508	1.16 (0.98–1.38)	0.092
Perceived health status				
Healthy	1		1	
Normal	1.04 (0.93–1.17)	0.460	1.26 (1.08–1.47)	0.004
Unhealthy	1.15 (0.99–1.34)	0.063	1.56 (1.24–1.97)	<0.001

* Multinomial logistic regression P-value

## Discussion

This study demonstrated that 33.0% of Korean women underestimated their weight status and 12.1% overestimated their body weight status relative to their actual BMI category. Despite highly prevalent misperceptions of weight status, especially in older women, the patterns of misperception varied among different age groups: greater underestimation occurred as age increased, and greater overestimation was found in younger age groups, irrespective of their actual BMI deciles and categories. After controlling for possible covariates, age was found to be the most important predictor of weight misperceptions in the opposite direction thereof (i.e., older women were more likely to underestimate body weight status and few overestimated it).

Several studies have suggested that weight perceptions are related to perceived susceptibility, which acts as a starting point for attempts at weight control [38, 39] and is more strongly correlated with weight loss attempts than actual weight status [21, 24, 40]. Indeed, women with overweight status who do not perceive that they are overweight have been found to be less likely to show interest in losing weight and to adhere to weight reduction behaviors [21, 41]. Thus, especially in older women with overweight/obesity, which accounted for more than 50% of women aged 60 years or more in the present study, underestimation of weight status may seriously prohibit the initiation of weight loss therapy or lifestyle changes to lose weight. As opposed to older women, younger women tended to be underweight and to overestimate their weight status. About 13.5% of women aged 20–29 years old had a BMI of <18.5Kg/m^2^, and 18.7% overestimated their weight status, especially among underweight or normal weight women.

While age has been shown to be related with over/under-perception of weight status in several studies [23, 25, 26, 34, 42, 43], these previous studies considered only one direction (either overestimation or underestimation), and the strength of the associations between age and misperception of weight was not strong. In this study, age was highlighted as the most strongly associated factor with misperception of weight in both directions, with significant linear trends therein. Previous studies showed that normal weight women who overestimate their weight status have been found to report more unhealthy weight-related behaviors [38, 44]. In addition, considering that weight misperception can elicit adverse psychological outcomes in various ways, especially in women who overestimate their weight status [45–47], younger women, the majority of whom were under or normal weight (more than 80% of women in their 20s and 30s in this study) and had the highest proportions of overestimation, would be a high risk group for not only unnecessary weight control and unhealthy weight-related behaviors, but also psychological problems.

Unlike Western populations where overestimation is less common and underestimation in women with overweight/obesity is a more pressing issue due to increasing obesity across populations that have “normalized” overweight and obesity [42, 43, 48], young Asian women perceived themselves as being overweight more and attempt weight control more than women in other countries [24]. Traditional Koreans considered being overweight as ideal feminine beauty and thinness as negative. However, traditional values regarding body image have changed fast, such that now a thin body is ideal [14]. In Korea, the strongest preferences for thinness were recorded for young adult females, compared to other countries [14, 49]. Considering a higher drive for thinness and more societal influence on body image toward younger women than older women [50], especially from peer groups [16], changing social norms regarding ideal body image needs to be achieved, along with promoting correct weight perceptions. Otherwise, for older women who might retain the traditional Korean ideal body image and may have different motivations for weight control [17], correcting weight perceptions and educating them health consequences of overweight/obesity could potentially motivate older women to achieve normal weight status.

Among all of the participants in this study, polarization of weight perceptions was observed (i.e., underestimation in women with overweight/obesity and overestimation in normal/underweight women). In general, self-reported weight is likely to be underreported, especially in individuals with overweight and obesity and in older women [51–53]. Therefore, the proportions of individuals with overweight or obesity could have been underestimated in this study population, especially among older women considering the higher misperceptions and the high prevalences of overweight and obesity in this age group. Comparing the prevalences of overweight and obesity in this study with results from the Korean National Health and Nutrition Examination Survey (KNHANES), in which height and weight were measured by trained medical staff [5, 54], we found that the prevalence of obesity in this study were lower, suggesting underestimation. Despite inaccuracy, several studies have compared BMI calculated by self-reported height and weight and perceived obesity, just as we did [26, 43, 48], suggesting that BMI from self-reported height and weight may be valuable to identify perceived weight status in comparison with reported weight.

Several limitations to the present study could influence the interpretation of our findings. The self-report bias for weight and height has already been discussed. Compared with other nationwide studies [5, 54], we observed a lower prevalence of overweight/obesity, as was discussed above, suggesting imprecise BMI estimation based on self-reported height and weight. In addition, although the proportion of underweight and overweight in terms of weight perception would be comparable to the results of the 2016 KNHANES and K-Stori, the proportion of those who perceived themselves as normal weight was higher and that of obesity was lower in this study (data not shown). A possible explanation for the difference in the weight perceptions may relate to the different study purposes of the KNHANES and the K-Stori, along with different sampling schemes (e.g., representation of total Korean population and representation of women in each life cycle, respectively). Second, BMI to define overweight and obesity cannot consider body fatness, and thus, may misclassify physically fit individuals with dense muscle mass as overweight. However, previous studies have shown that BMI is strongly correlated with body fat percentage [55]. Third, the response rate of this study was only about 40%, and this might introduce non-response bias. However, recent nationwide surveys conducted in Korea have reported similar response rates (33~70%) [56–58], suggesting a common limitation among surveys in Korea. Fourth, despite having been given weights in consideration of the complex survey design, sampling ratio, and non-response rate, we utilized unweighted values due to the exclusion criteria we applied. Thus, the initial weights might not be applicable, and it might hinder the generalizability of the study results.

Despite these limitations, this study provides novel findings that suggest age is the most important factor associated with weight perceptions among Korean women, affecting both underestimation and overestimation of weight status. The different direction of weight perceptions according to age may suggest the need for a stronger emphasis on body image for young women [43] and a stronger effect of mass media on younger women in regards to ideal body image and weight control [18]. Further research that links objective BMI, reported BMI, perceived weight status, weight control behaviors associated with further psychological consequences, and health outcomes based on societal stigma toward obesity is needed to support the implications of our findings.

## Conclusions

In the present study, we found age group differences with regard to perceived weight status: higher underestimation in older women and higher overestimation in younger women. Moreover, age showed the strongest association with both underestimation and overestimation of weight perceptions in women.
